# Heart valve disease: investigation by cardiovascular magnetic resonance

**DOI:** 10.1186/1532-429X-14-7

**Published:** 2012-01-19

**Authors:** Saul G Myerson

**Affiliations:** 1 Consultant Cardiologist, John Radcliffe Hospital, Honorary Senior Clinical Lecturer, University of Oxford Centre for Clinical Magnetic Resonance Research, Oxford, UK

**Keywords:** Cardiovascular Magnetic Resonance, Valve disease, Flow quantification

## Abstract

Cardiovascular magnetic resonance (CMR) has become a valuable investigative tool in many areas of cardiac medicine. Its value in heart valve disease is less well appreciated however, particularly as echocardiography is a powerful and widely available technique in valve disease. This review highlights the added value that CMR can bring in valve disease, complementing echocardiography in many areas, but it has also become the first-line investigation in some, such as pulmonary valve disease and assessing the right ventricle. CMR has many advantages, including the ability to image in any plane, which allows full visualisation of valves and their inflow/outflow tracts, direct measurement of valve area (particularly for stenotic valves), and characterisation of the associated great vessel anatomy (e.g. the aortic root and arch in aortic valve disease). A particular strength is the ability to quantify flow, which allows accurate measurement of regurgitation, cardiac shunt volumes/ratios and differential flow volumes (e.g. left and right pulmonary arteries). Quantification of ventricular volumes and mass is vital for determining the impact of valve disease on the heart, and CMR is the 'Gold standard' for this. Limitations of the technique include partial volume effects due to image slice thickness, and a low ability to identify small, highly mobile objects (such as vegetations) due to the need to acquire images over several cardiac cycles. The review examines the advantages and disadvantages of each imaging aspect in detail, and considers how CMR can be used optimally for each valve lesion.

## Review

Cardiovascular magnetic resonance (CMR) has unique capabilities which can greatly benefit the assessment of the patient with cardiac valve disease. While echocardiography (echo) remains the major imaging modality for assessing valve disease, there are many areas where CMR provides 'added value' to existing assessment and can complement the echo assessment. CMR can also provide a comprehensive 'stand-alone' assessment in some situations, delivering optimal assessment of patients using a combination of several techniques. These include quantifying the severity of the valve lesion, determining aetiology, examining the consequences for the relevant ventricle, and assessment of the surrounding anatomy (e.g. aortic root). Additional information on great vessel anatomy and the presence of myocardial scar (infarction) can also be clinically useful. The modality is used best by harnessing the advantages it brings, rather than attempting to replicate echocardiography or x-ray computed tomography (CT). This review will highlight the optimal use of CMR in valve disease, highlighting the strengths of the technique and also the potential pitfalls when assessing patients with valve disease.

## The advantages of CMR in valve disease

### Valvular function & anatomy with unlimited imaging planes

**Figure 1:**
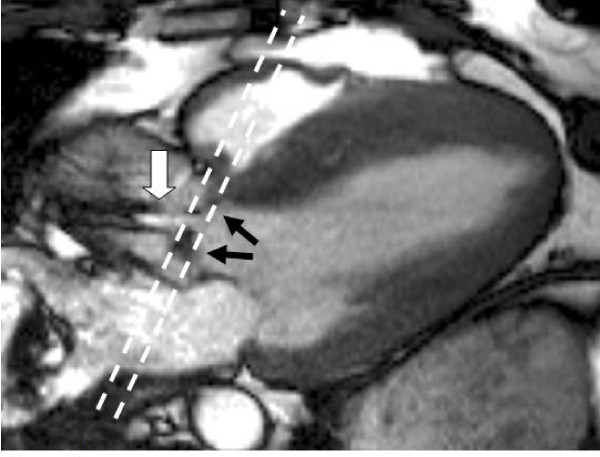
**SSFP sequence in the LV outflow tract view in systole showing restricted aortic valve leaflets (black arrows) and a high velocity jet of aortic stenosis (white arrow), with high signal (white) from the more stable core surrounded by low signal (black) due to shear and turbulence**. Parallel dashed lines indicate the slice position for imaging the valve tips for assessment of valve area (Figure 6).

Most morphological and functional information is obtained using cine CMR sequences, particularly steady state free-precession (SSFP) sequences with their high contrast between blood pool and surrounding structures (Figure 1). These have largely replaced spoiled gradient echo sequences, though the latter remain useful on occasions for visualising the extent of flow disturbance in selected cases. The ability to image in any plane allows clear views of all four cardiac valves and their inflow/outflow tracts, irrespective of thoracic anatomy or difficult cardiac anatomy. This is particularly useful for right-sided valves which can be challenging to visualise with echo, particularly the pulmonary valve. An additional advantage is that it facilitates direct measurement of valve orifice area for stenosed valves by planimetry rather than calculation [1], and the same technique can occasionally be used for the assessment of regurgitant orifices if required. The anatomical information from CMR cine images can be at least as good as transoesophageal echocardiography, and valvar anatomy and function can often be visualised well with cine images, along with the mechanism of regurgitation, particularly with thin (4-5 mm) slices. There are however several limitations of CMR cine assessment, including a relatively thick imaging slice (typically 5-8 mm) resulting in partial volume effects, and the need to acquire cine images over several cardiac cycles which results in sub-optimal visualisation of small or more chaotically mobile objects such as vegetations. The thin nature of cardiac valves (typically 1-2 mm) makes them particularly prone to partial volume effects due to the slice thickness of CMR images. Care is therefore required in placing image slices perpendicular to the valve plane to minimise these effects and in minimising slice thickness to 4-5 mm, but some aspects of finer valve anatomy may be too difficult to visualise well with CMR. Furthermore, for accurate assessment of the stenotic/regurgitant orifice, positioning the image slice precisely at the valve tips is important and misalignment may result in significant error. Multiple parallel thin image slices in the plane of interest can help to locate the one slice at the optimal position of the valve tips.

**Figure 2:**
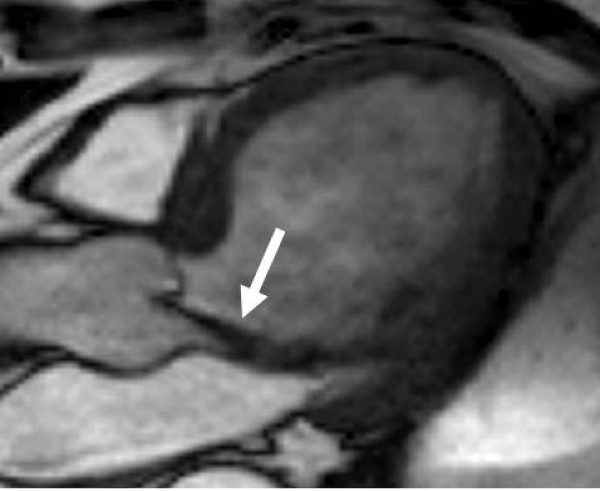
**LVOT view in diastole showing jet of eccentric aortic regurgitation (arrow) visualised by the low signal on SSFP sequences, due to spin-dephasing caused by shear and turbulence**.

The visual assessment of turbulent flow in stenotic or regurgitant flow jets is also feasible with the cine sequences described above, through visualisation of signal voids due to spin dephasing in moving protons [2]. Flow related signal loss seen on SSFP images occurs where voxels span a range of velocities, notably in the shear layers that can surround the more coherent jet core. The location and direction of regurgitant or stenotic jets can be assessed (Figure 2), which can provide valuable information about the valve lesion. In regurgitant jets, the width of the jet origin and/or the vena contracta provide useful information, with milder leaks having narrower jets in general. Lastly, the cine-visualised flow jets can also assist in planning the placement of subsequent velocity-encoded images. Signal voids seen on SSFP imaging are however substantially related to the acceleration of blood rather than the velocity alone, and may underestimate the degree of flow disturbance when assessing the degree of regurgitation. Narrow (mild) jets may be difficult to visualise due to the lack of shear layers at the edge of the jet. Gradient echo sequences are more sensitive than SSFP sequences for evaluating the presence and magnitude of turbulent jets [3], and this sensitivity is increased with lengthening echo time [4,5]. Assessing the severity of regurgitation with visual assessment of cine images requires care and caution however, as the technique is subject to slice positioning, partial volume effects, the insensitivity of SSFP sequences, and to other sequence parameters. This method can provide an approximate guide to the degree of regurgitation, and distinguishing mild and severe regurgitation is feasible, but finer differentiation of severity is rarely possible.

### Accurate and reproducible ventricular volumes, function and mass

Accurate measurement of left and right ventricular volumes, function and mass are vital for assessing the impact of valve lesions on the ventricles. Excessive dilation or reduced ventricular function are strong indicators of a poor prognosis [6], and reliable measurement is important. CMR is the most accurate and reproducible technique for assessing both left and right ventricular volumes & mass [7-9], and newer steady-state free-precession sequences appear to be even more accurate than older gradient echo cine sequences [10,11]. RV volumes are particularly useful as these are difficult to achieve by other methods, though accurate measurement is more difficult than for LV volumes. The role of left ventricular (LV) mass in valve disease has not been studied as extensively as volume, possibly due to the inaccuracies of measurement by M-mode or 2-dimensional echo [12], and LV mass may become a useful measure in the future, particularly for patients with aortic stenosis. Reproducibility is important for serial assessment of ventricular size, as patients with valve lesions are often monitored for many years if asymptomatic before symptoms and/or ventricular deterioration occur. CMR is highly reproducible [13], and being a 3-dimensional technique, is more sensitive to changes than one or two-dimensional LV diameters [12]. CMR is also less prone than echocardiographic diameters to variations in measurement position, which can occur, despite standard guidelines [14]. The accuracy of CMR is, however, dependent on correct placement of the basal ventricular image slice, and careful contour placement during post-processing is crucial, with correct differentiation of atrial and ventricular chambers, especially for the right ventricle. Significant error can occur if the basal slice is incorrectly included/excluded from ventricular volumes. Post-processing software that includes long axis visualisation of the valves to ensure appropriate slice inclusion significantly aids accuracy. CMR-derived ventricular stroke volumes can also be used to quantify mitral and tricuspid regurgitation, either in conjunction with flow measurement or with volume data alone if the valve regurgitation is isolated [15] - see below, but the issues about accuracy of contour placement still apply.

### Flow and velocity quantification

**Figure 3:**
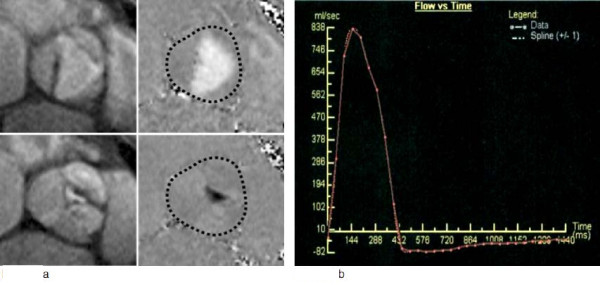
**Through-plane phase contrast velocity mapping for flow quantification of aortic regurgitation at the valve tips**. Left: magnitude (anatomical) images in systole (top) and diastole (bottom). Right: corresponding phase (velocity) images at the same phase; forward flow in white, regurgitant flow in black; dotted line represents region of interest for post-processing of velocity data. **Resulting flow-time graph after integration of velocity in each voxel over one cardiac cycle, demonstrating forward flow above the line and regurgitant flow below the line**. The area under the curve represents the volume of flow, and can be calculated by the software.

The ability to quantify flow directly using through-plane phase contrast velocity mapping [16] is a unique advantage of CMR, and does not rely on calculation from complex equations, as echo or invasive catheterisation techniques require. The technique exploits the property of protons moving in a magnetic field gradient, in which they acquire a shift in the phase of their rotational spin as compared with stationary protons, and the magnitude of this phase shift is proportional to their velocity. By producing images from the phase information, velocity can be measured [17], and is visually displayed in greyscale images (Figure 3a). Flow is derived from through-plane velocity maps by integrating the velocity of each pixel and its area over time, typically a single cardiac cycle (Figure 3b). CMR flow measurement shows good accuracy in *in-vitro *studies and it correlates well with invasive *in-vivo *measurements [18-22]. *In vivo *studies are hampered by the lack of a true 'gold standard' technique for comparison - invasive measures of flow rely on complex calculations and assumptions which may not hold true. The temporal resolution of CMR flow measurement is typically 25-45 msec, which is lower than for continuous wave Doppler velocity measurements in echo, but is good enough for most flow and velocity measurements. Flow measurement is however critically dependent on a homogenous magnetic field, and ensuring the image slice is at the magnet isocentre is important for minimising error. Despite this, phase offset errors due to eddy currents in the magnetic field can still occur, affecting background flow measurements. These are likely to be worse with newer breath-hold sequences due in part to faster gradient switching, and these sequences appear to have poorer accuracy than non-breath-hold sequences [23,24]. Background flow correction using phantoms can correct the majority of the error [23,24], and is important for accuracy [25]. The majority of the validation work has been carried out using older non-breath-hold flow sequences, and these are recommended for accurate flow measurement due to the lower background offset error and better validation, particularly if phantom correction is not used. The background offset errors are also worse in very oblique imaging planes [25], and using imaging planes closer to the transverse body plane is helpful [25], where feasible.

**Figure 4:**
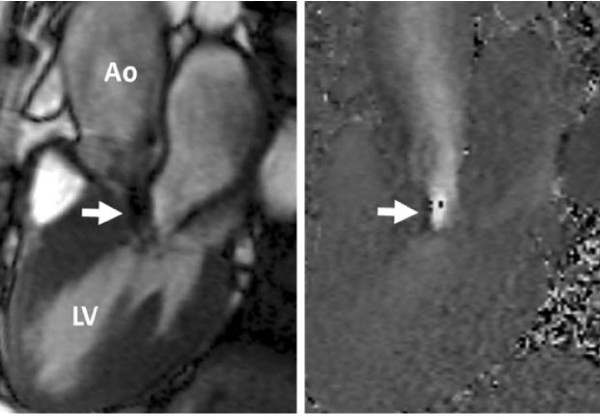
**Example of in-plane phase contrast velocity mapping in the LV outflow tract in a patient with dynamic outflow tract obstruction**. Left: cine image; right: corresponding phase image from in-plane velocity mapping. The highest velocities can be visualised in the outflow tract (arrowed), below the aortic valve. LV = left ventricle; Ao = aorta.

Velocities can also be assessed with either 'through-plane' velocity mapping (as used for flow above) or 'in-plane' phase contrast sequences, measuring velocity within the plane of the slice (Figure 4). The in-plane sequences can demonstrate the origin and direction of a jet, and can be useful for visualising the site of stenosis and measuring velocity along the course of a jet, or can assist in planning the subsequent perpendicular or 'through-plane' slice. In-plane sequences may be less accurate however for measuring velocity in a stenotic lesion, particularly peak velocity, for a number of reasons. Due to the image slice thickness (typically 5-7 mm relative to a stenotic jet width of 2-4 mm), they are subject to partial volume effects, in which several velocities occur within a single voxel and an averaged phase shift is measured [26]. The temporal resolution, while reasonable (typically 20-25 msec), is low when compared to fast-changing jet velocities (by comparison, continuous wave Doppler echo temporal resolution can be ~2 msec), and the true peak velocity may be missed. Lastly, the accuracy of CMR velocity measurement in high velocity jets is reduced, particularly with velocities above 3.5-4 m/sec [21,22]. This is due to signal loss from turbulence [26], and phase shift errors due to fast acceleration and intravoxel dephasing. Utilising sequences with a very short 'echo-time' (~2 msec) can reduce these errors [22] and future applications may use these 'ultra-short' echo time sequences. Narrow, high velocity flow jets (e.g. severe aortic stenosis) are thus especially affected, and the most severely stenotic jets may be too narrow and turbulent for accurate velocity measurement. Through-plane velocity mapping sequences may reduce some of the errors, particularly partial volume effects, as they take advantage of the better within-plane image resolution (typically ~1 mm), to cope with narrow jets. They are thus the preferred method for accurate velocity measurement, though the acceleration & turbulence errors still apply and the temporal resolution is similar. The highest velocity narrow jets may still be too difficult to measure however. Through-plane velocity measurement relies on the plane being placed at the site of maximal velocity (if peak velocity is desired), which is why in-plane velocity mapping to guide placement of the through-plane image can be helpful, unless the site of maximal velocity is predictable. Fortunately, background phase offset errors only result in a small change in the velocity of individual voxels (as opposed to moderate differences when summed for flow quantification), so do not substantially affect velocity measurement.

In addition to flow quantification, recently developed 3-dimensional in-plane CMR flow sequences can measure velocities in 3 dimensions simultaneously [27,28], allowing the visualisation of complex flow patterns. Future work may examine the utility of this technique for valve lesions and other areas of clinical utility.

## Left-sided valve lesions

### Aortic stenosis

**Figure 5:**
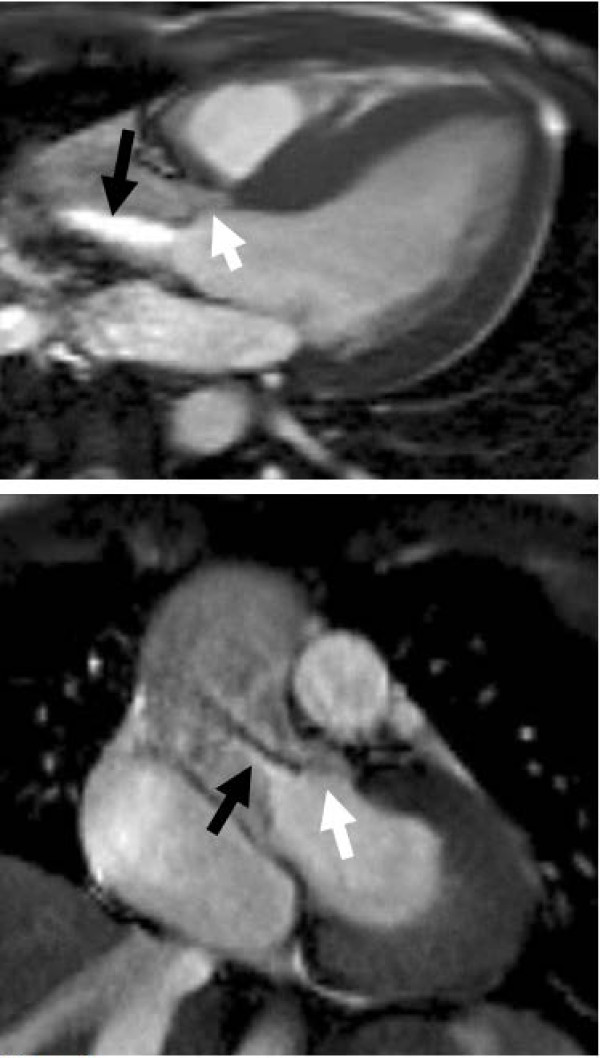
**Aortic stenosis in a bicuspid aortic valve: standard left ventricular outflow tract (LVOT) view (top) and 'coronal' LVOT view (bottom), acquired perpendicular to the standard LVOT view through the valve tips**. Note the domed leaflets with restricted tips typical for congenital stenosis (white arrows) and the high velocity jet (black arrows).

**Figure 6:**
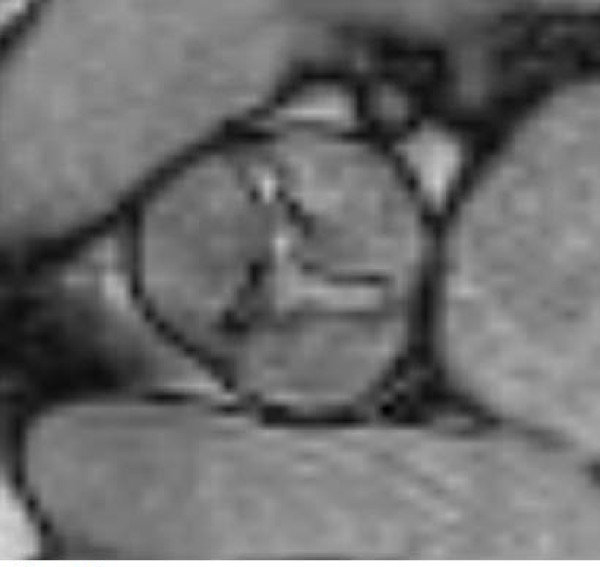
**Through-plane SSFP image in systole through the valve tips in a patient with aortic stenosis**. The tips are outlined by dark (low signal), partly due to signal loss from shear. The orifice area can in this case be measured directly by planimetry, but this should not be attempted if the outlines are unclear on the available cine images.

The assessment of aortic stenosis is enhanced with CMR through accurate assessment of the anatomy of the valve and aortic root, quantification of LV mass and function to indicate the precise effect on the LV, and measurement of the velocity of the stenotic jet where this is difficult with echo. The excellent visualisation of anatomy provided by CMR allows accurate evaluation of the severity of aortic stenosis [29], usually starting with standard and 'coronal' LV outflow tract views (Figure 5), from which a good qualitative assessment of the valve can be made. Direct planimetry of the aortic valve orifice is the most useful technique for quantifying stenosis severity, achieved by placing an imaging plane through the valve tips in systole (Figure 6). It is important however to ensure the image slice is thin (4-5 mm) and precisely at the valve tips, and acquiring multiple parallel thin slices parallel to the valve orifice may aid in identifying the true orifice [30]. This technique agrees well withmeasured aortic valve area on transoesophageal echocardiography [30-32] and also with estimated valve area from the continuity equation [31,32]. Trans-valvar velocity can be measured withvelocity mapping [33,34], though peak velocity may be less accurate (often underestimated) compared to continuous wave Doppler echo, due to the small width of very high velocity jets (partial volume effects), lower temporal resolution, and artefacts from turbulent jets, as indicated earlier. For these reasons, direct planimetry of the valve orifice area may be a more reliable assessment of aortic stenosis with CMR. It has been suggested that the continuity equation could be used with CMR to estimate valve area [35], with direct measurement of the LVOT area being an advantage over echo. However, this is unnecessary when CMR can directly measure the valve area itself, and the continuity equation relies on multiple measurements and an equation to calculate (rather than directly measure) the valve area, all of which increase the potential for error.

Despite the limitations, CMR measurement of velocity is advantageous in angulated roots where correct echo beam alignment with the stenotic jet is difficult. In addition, many stenotic jets are not parallel to the LV outflow tract, and are also inaccurately assessed with echo, even when outflow tract visualisation is good. In-plane velocity mapping in the outflow tract is useful to identify the location of maximal velocity - this is usually just distal to the valve tips in valvar stenosis. This can be followed with through plane velocity mapping in a plane perpendicular to the direction of flow, positioned at the identified location of maximal velocity (Figure 1). This combination reduces partial volume effects while ensuring the peak velocity is measured, and mean velocity can also be assessed from the through-plane velocity measurements. Ensuring the correct slice position for flow measurement is important for accuracy; and although this image appears similar to that through the valve tips, the position of maximal velocity (the vena contracta) usually lies a few millimetres distal to the valve tips, so the images may not be in identical locations.

Other advantages in aortic stenosis include the ability to differentiate sub-valvar and supra-valvar stenosis, which are easily visualised with CMR cine imaging, and the site of velocity acceleration can be accurately located with in-plane velocity mapping. CMR can also accurately assess the ascending aorta, which may be dilated, particularly with bicuspid aortic valves, and may alter surgical management either at the time of valve replacement or by indicating that root ± valve replacement are required due to excess aortic dilation.

Highly accurate LV mass measurement provides a more precise and sensitive measure of the effect of aortic stenosis on the left ventricle than measuring myocardial wall thickness. LV mass has been a poorly examined parameter in aortic stenosis, likely due to the inaccuracies of echocardiographic M-mode measurement, and CMR-derived LV mass may prove to be a useful tool but needs further examination. Late gadolinium enhancement imaging in patients with aortic stenosis has shown patchy mid-wall enhancement in up to a third of patients with severe stenosis, usually in conjunction with significant LV hypertrophy, and often in the basal lateral wall [36]. This likely reflects focal areas of fibrosis, which have also been shown in autopsy studies [37]. Early reports have shown that this is associated with a worse prognosis [38]. Future CMR techniques may include the assessment of diffuse fibrosis using T1 mapping and other CMR techniques, and this is likely to be an exciting area of development.

### Aortic regurgitation

**Figure 7:**
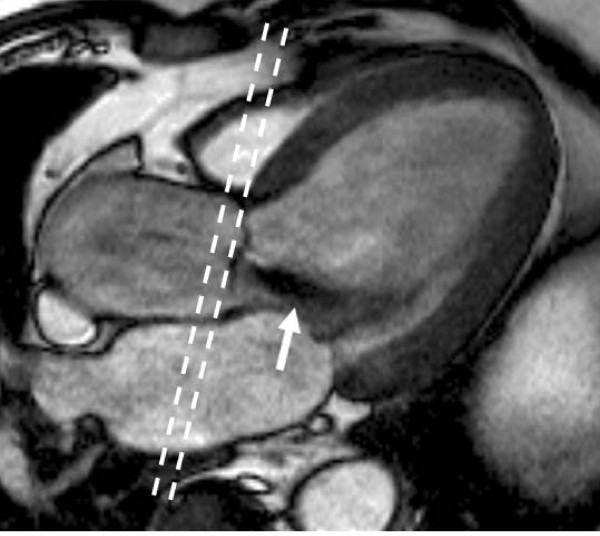
**LVOT view in diastole showing turbulent jet of aortic regurgitation (arrow) and position for through-plane velocity mapping image (dashed lines) to quantify aortic flow**.

**Figure 8:**
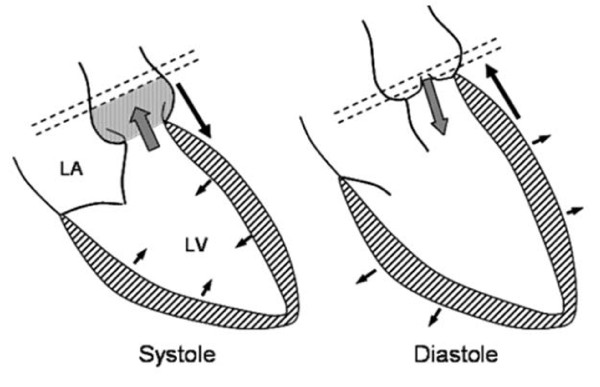
**(taken from the Oxford Handbook of CMR, OUP) *Mechanism for potential underestimation of aortic regurgitation***. The gap between the valve and the image plane for flow mapping expands in systole from a combination of movement of the aortic valve towards the apex and elastic expansion of the aortic sinuses and root. Blood entering this space in systole (grey stippled area) returns to the LV in diastole (via a regurgitant valve) without passing through the image plane (dashed lines), and thus may not be measured.

The advantages of CMR in aortic regurgitation are quantitation of the regurgitation and of LV volumes and function, particularly for serial measurement. Aortic regurgitation is difficult to quantify with echocardiography, which mostly relies on qualitative and semi-quantitative measures of severity. CMR can accurately quantify the amount of regurgitation using flow mapping, and derived values such as regurgitant fraction (regurgitant volume/forward volume × 100%) can be obtained. Imaging the valve occurs in much the same way as for aortic stenosis, using long axis LVOT views (Figure 7), from which qualitative visualisation of the regurgitation can be performed (Figure 2). Flow can be measured by placing the imaging slice for flow mapping just above the aortic valve, quantifying both forward and regurgitant flow per cardiac cycle [20,39,40] (Figure 3). Positioning the imaging slice just above the valve (rather than in the mid-ascending aorta) is important (Figure 7), despite the higher and more turbulent forward velocities encountered here, as underestimation of the regurgitation can occur otherwise [18]. This is due to a number of factors, including movement of the valve towards the LV apex during systole which allows blood to flow into the gap between the imaging plane and the valve. This blood may return to the ventricle during diastole and not flow through the imaging plane, thus being lost to measurement (Figure 8). This tendency is exacerbated by several factors: aortic distension during systole, a dilated aortic root (resulting in greater volume between the imaging plane and the valve), and vigorous longitudinal contraction of the LV (common in significant AR). Placing the plane closer to the valve minimises these errors, though avoiding the very highest turbulence at the valve tips themselves is wise. An ideal flow sequence would incorporate slice tracking to minimise these errors, which tracks the aortic valve and moves the imaging slice accordingly, but this involves complex software programming and has only been performed at a single centre so far [41].

The accuracy of aortic regurgitation quantification using CMR through-plane velocity mapping is excellent when compared to in-vitro studies [42] or in-vivo CMR measurement using the difference between ventricular volumes [20], and it correlates well with angiographic or echocardiographic grades of severity [18,20,40]. As it remains the only technique capable of true in-vivo quantification of aortic regurgitation (without calculation), there is no 'Gold standard' for comparison of accuracy. Reproducibility is also good, both for inter-study and intra/inter observer comparisons [39,40]. The technique is however subject to the same potential problems as all flow techniques, highlighted earlier (under 'flow and velocity quantification'), and care is required to ensure good accuracy of the measurements. In particular, non-breath-hold flow sequences are recommended for their lower background flow offset errors. Quantifying AR with CMR has recently shown a good ability to predict symptom development and the need for valve replacement surgery in the near future [43], with a regurgitant fraction > 33% providing the optimal threshold for identifying patients likely to require surgery within a few years. Given the difficulty in timing valve replacement surgery in patients with severe AR [44], this may become a valuable tool in clinical management, with the potential to identify suitable patients for early surgery. The potential improvement in outcome requires confirmation in a clinical trial however.

AR quantification can also be achieved by a comparison of the differences in LV and RV stroke volume from cine imaging alone [45], but this is less direct and relies on the lack of any other valve regurgitation or shunt. It also assumes accurate contour placement for volumetric assessment, and inaccuracies in measuring any of the four sets of contours (LV and RV in both diastole and systole) can result in significant errors. Careful contour placement is therefore required for this technique, particularly for the difficult RV contours. It is however a useful technique when flow quantification cannot be performed, or as an internal validation of the flow technique. An approximate assessment of the severity of aortic regurgitation can also be obtained by visualisation of the signal void of the regurgitant jet on cine imaging. A narrow jet width at the origin suggests lower degrees of regurgitation, while a wide jet, particularly with a core of high signal from laminar flow, suggests more severe regurgitation. This method is subject to many potential errors however (indicated earlier), and is not recommended for accurate evaluation.

Accurate LV volumes with CMR can aid clinical assessment of the impact of AR, and the high reproducibility is particularly useful for serial assessment, which is important for the management of a condition that has a long asymptomatic phase. CMR-derived LV end-diastolic volumes have also shown some ability to predict the onset of symptoms or other indications for valve surgery [43], and although less strong than quantifying the regurgitation itself, it can provide a useful adjunct in predicting outcome. CMR can also provide a detailed assessment of aortic root anatomy, which can assist in identifying the cause of the regurgitation, and/or whether the root needs replacing at the time of valve replacement surgery.

Taken together, the many CMR techniques useful in AR, including regurgitation quantification, LV volumetric assessment and aortic root anatomy, make CMR the optimal tool for comprehensive assessment.

### Mitral regurgitation

**Figure 9:**
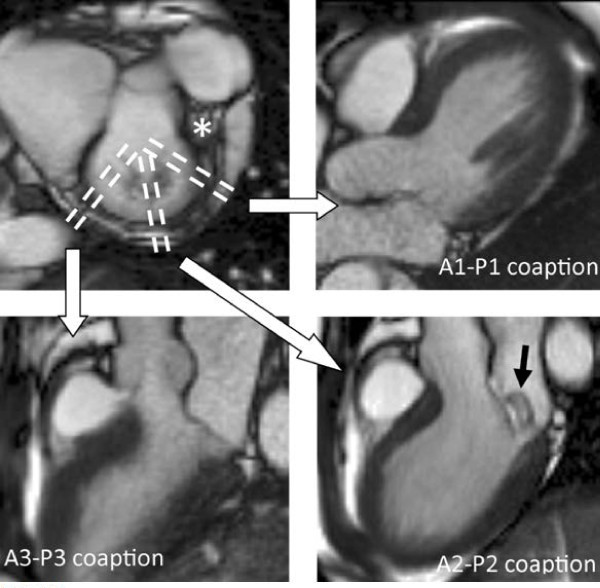
**Imaging the mitral valve segments - a modified approach adapted from reference 47**. A basal short axis slice through the mitral commissure is used (top left) to orientate the subsequent image slices for each of the segments. The left atrial appendage is identified by the asterisk (*). Cine sequences are then acquired perpendicular to the mitral commissure for each of the 3 scallops (parallel dashed lines). Systolic frames from the resulting images are shown here (top right and bottom images). In this patient, there is isolated prolapse of the posterior leaflet P2 segment (bottom right, black arrow), but other segments have normal coaption (top right and bottom left).

As for aortic regurgitation, the main advantages of CMR in mitral regurgitation are in quantitative assessment of both the regurgitation, and ventricular volume and function. CMR can also assess leaflet morphology and valve function, particularly utilising the free choice of image planes to characterise the aetiology of the regurgitation in this complex valve. CMR has good agreement with trans-oesophageal echocardiography for assessing mitral valves for repair [46], though the slice thickness can result in partial volume errors more frequently than echocardiography, which has a very narrow beam width. A good method is to place multiple cine images perpendicular to the mitral valve commissure, facilitating assessment of the individual scallops/coaption, and can identify the site of localised prolapse/regurgitation [47]. This technique can be modified by placing three slices specifically across the commissure, perpendicular to the edge of the leaflet at each of the scallops (Figure 9). This results in reduced partial volume effects in the A1/P1 and A3/P3 views particularly, while also being marginally quicker. Despite these good techniques, transoesophageal echocardiography is likely to remain the optimal investigation for leaflet assessment, due to its superior leaflet detail and potential 3-dimensional images. CMR could be used if trans-oesophageal echocardiography wasn't possible, if doubts remained after the echocardiogram, or if other aspects required assessment by CMR and all could be investigated by a single CMR scan. CMR can also be valuable in assessing the regurgitant orifice using thin (4-5 mm) slices parallel to the mitral annulus, carefully placed through the mitral tips in systole [48]. In some patients this can provide direct visualisation and measurement (if required) of the regurgitant orifice area (rather than calculated area from echocardiography), but the complex shape and motion of the valve mean this is not feasible in all patients.

**Figure 10:**
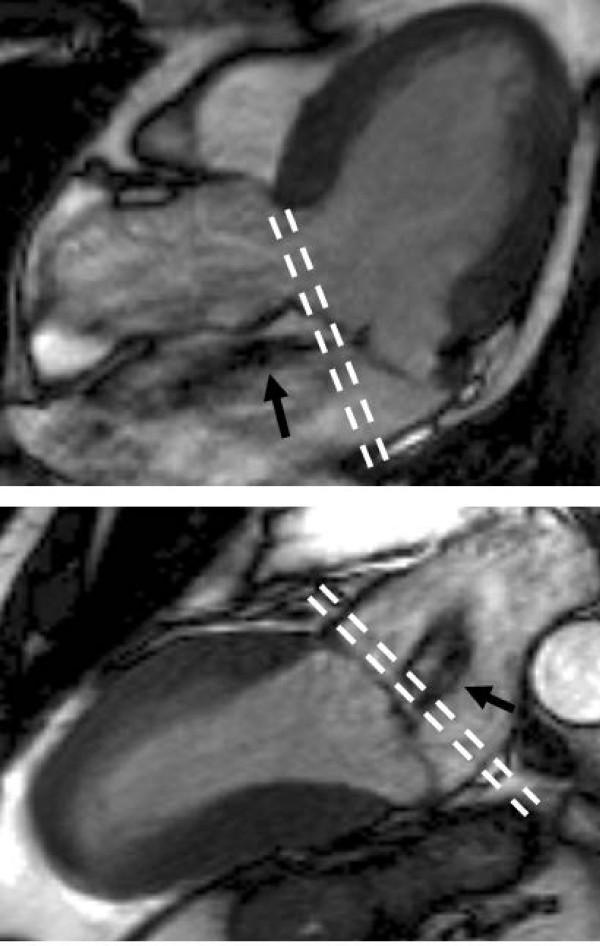
**Location for direct assessment of mitral regurgitation flow using through-plane velocity mapping**. Slice position indicated by dashed lines in LVOT view (top) and VLA view (bottom), perpendicular to the regurgitant jet (arrowed).

Quantification of mitral regurgitation is usually performed indirectly, mostly by subtracting aortic systolic flow (measured by aortic flow mapping described above) from LV stroke volume [29]. This relies on a combination of two different MR techniques and increases the potential for measurement error, so care is required with the flow sequence and LV contours, as previously highlighted. Mitral regurgitation can also be quantified by comparing the difference in ventricular stroke volumes, as for aortic regurgitation, but the same limitations apply, including the assumption of a single valve lesion and the need for accurate contour placement. The quantification of mitral regurgitation correlates well with echocardiographic and angiographic assessmentand has good reproducibility [49-51]. The potential clinical utility of quantification is shown by the good ability to predict the future need for surgery in asymptomatic patients [52], similarly to aortic regurgitation, with a regurgitant fraction of 40% providing the optimal threshold for MR. Although this prognostic ability was not quite as strong as for aortic regurgitation, it is significantly greater than echocardiographic semi-quantitation using the proximal isovolumetric surface area (PISA) technique for estimating mitral regurgitant orifice area [53]. Quantitative CMR measures may become a valuable clinical tool to identify suitable patients for early valve surgery, as for aortic regurgitation, though again clinical trials are required to determine if this potential for identifying patients for early surgery translates into improved clinical outcomes. Direct quantification of the regurgitation using CMR is possible using through-plane flow mapping on the atrial side of the mitral valve [54] (Figure 10), but is difficult due to the highly mobile valve and often eccentric, mobile jets of regurgitation. Despite these difficulties, it has reasonable agreement with indirect quantification and can provide an alternative method. Atrial fibrillation is more common in patients with mitral regurgitation, and this can reduce the accuracy of flow measurements [54], but is improved if the heart rate variability is low [54].

Similarly to AR, an approximate assessment of the severity of mitral regurgitation can be obtained by visualisation of the signal void of the regurgitant jet on cine imaging, with a narrow jet width suggestinglower degrees of regurgitation and a wide jet (particularly with a core of high signal) suggesting more severe regurgitation. However, the same limitations apply, and this method is only useful as a rough guide.

### Mitral stenosis

**Figure 11:**
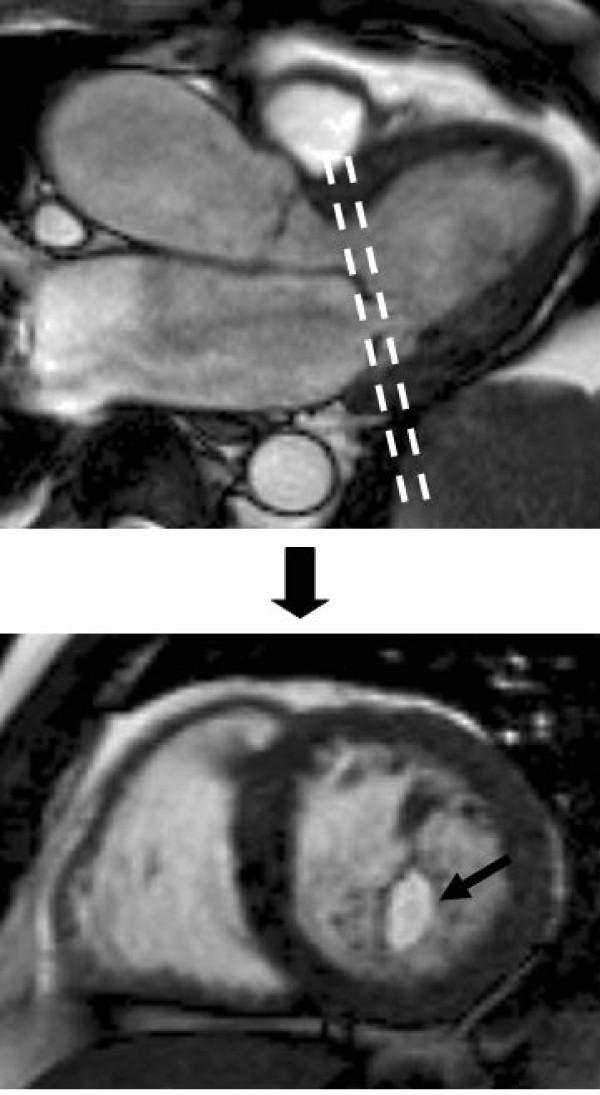
**Assessing mitral stenosis by direct planimetry of the mitral tips**. Top: diastolic frame from an LVOT view demonstrating slice position for subsequent imaging (dashed line). Bottom: resulting modified short axis view through the mitral tips in diastole, showing the easily visualized mitral orifice (arrow).

Echocardiography, particularly trans-oesophageal echocardiography, remains the first line technique for assessing mitral stenosis due to its excellent visualisation of the mitral leaflets. CMR can be helpful in selected cases however, with good visualisation of the restricted mitral leaflets, particularly on the LVOT view. Direct measurement of the orifice area can be performed in much the same way as for aortic stenosis, by placing an imaging plane at the mitral valve tips during diastole (Figure 11). The technique has good agreement with echocardiography [55], but care needs to be taken in positioning the plane at the tips in order to obtain an accurate valve area, and multiple parallel thin slices may be helpful. Diastolic flow and velocity can also be measured in this image plane, and pressure half time calculated as for echocardiography [56], though the frequency of atrial fibrillation in severe mitral stenosis reduces the accuracy of the flow measurements [54].

## Right-sided valve lesions

### The pulmonary valve

The pulmonary valve and right ventricular outflow tract can be difficult to assess with echo, due to several factors. The location of the valve and outflow tract immediately behind the sternum makes it difficult to position the echo probe adequately to visualise the area. The qualitative echo assessment of pulmonary regurgitation is also less robust than for aortic regurgitation, and grading regurgitation severity can be difficult. Thirdly, the right ventricle can be difficult to assess, particularly for volumetric assessment, due to its unusual shape. CMR is therefore particularly valuable for assessing the pulmonary valve, with its combination of free choice of imaging planes, velocity & flow assessment, and accurate assessment of RV anatomy and volumes. Despite the very thin nature of the normal pulmonary valve, making it difficult to visualise with CMR, these other advantages are considerable, and CMR should be considered the 'Gold standard' for assessment of the pulmonary valve and RV outflow tract.

#### Pulmonary stenosis

**Figure 12:**
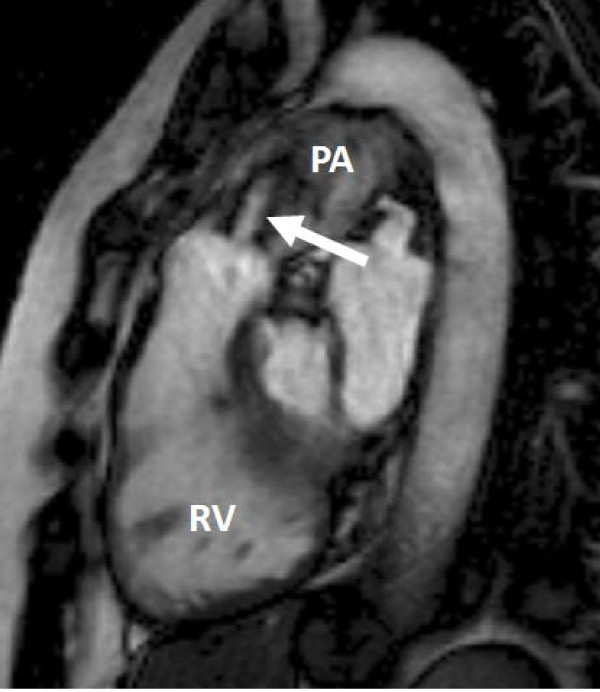
**Right ventricular outflow tract view showing fore-shortened right ventricle (RV) and a pulmonary valve with moderate-severe stenosis**. The high velocity jet of the stenosis can be seen (arrowed). PA = main pulmonary artery.

The excellent visualisation of the RV outflow tract with CMR facilitates easy identification of the site and severity of pulmonary stenosis. An RV outflow tract view (usually an oblique sagittal plane), including the proximal pulmonary trunk and a very fore-shortened right ventricle, is ideal (Figure 12). Care is required however to ensure the outflow tract remains in the plane during the whole cardiac cycle, due to the significant long axis motion of the RV. Acquiring a cine image in a more horizontal plane through the RVOT, perpendicular to the first (imperfect) RVOT view, can help plan a subsequent improved RVOT view throughout the whole cardiac cycle. A qualitative assessment of severity can be made from the cine views, by visualising the valve motion and stenotic jet. Quantitative assessment is similar to that for aortic stenosis, and direct planimetry of the valve orifice from a cine image through the valve tips is the preferred method for assessing severity. Peak velocity can also be measured, as for aortic stenosis, though has similar limitations, particularly for high velocity jets. Identifying sub-valvar and supra-valvar stenosis is straightforward with the long axis views through the outflow tract, and in-plane velocity mapping in these planes can be helpful in identifying the point of maximal velocity where doubt remains. Lastly, RV mass and function can be assessed to determine the effect on the RV, and any concomitant pulmonary trunk or branch artery stenosis can be identified with either a thin-slice SSFP anatomical stack or MR angiography of the pulmonary arteries.

#### Pulmonary regurgitation

Trivial or mild pulmonary regurgitation (PR) is common in normal subjects (~30% of the population [57]), but rarely of importance. More significant degrees of regurgitation are usually related to congenital heart disease, with the largest group being patients with repaired tetralogy of Fallot, who commonly have significant residual PR [58-60]. CMR has revolutionised the investigation and follow up of such patients, as it accurately assesses two important aspects - the quantity of regurgitation and RV volumes/function [61], and is the method of choice for examining PR in this patient group.

**Figure 13:**
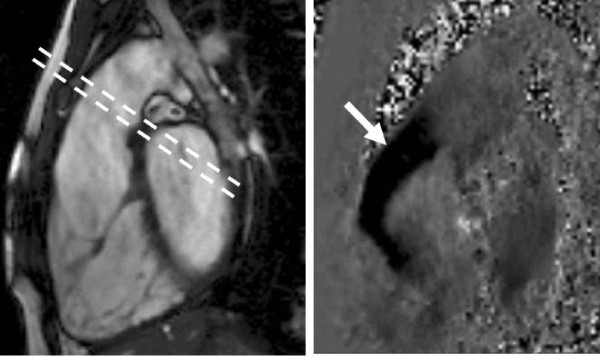
**Left: RV outflow tract view in diastole in a patient with repaired Fallot's tetralogy and severe pulmonary regurgitation**. There is almost no turbulence, due to the wide jet with mostly laminar flow. The flow can be visualized with in-plane flow imaging (right), where the wide regurgitant jet is seen in black (arrowed). The dashed lines on the cine image indicate the slice location for through-plane velocity mapping.

The assessment is similar in general to aortic regurgitation. RV outflow tract cine images can give an idea of anatomy, but as pulmonary pressures are lower than systemic, the degree of turbulence from PR is often lower and may be poorly visualised on cine images, especially SSFP (Figure 13). In-plane phase contrast velocity mapping sequences are better for visualising the regurgitant jet (Figure 13). Accurate quantification can be performed using through-plane velocity mapping, with the image slice placed just above the pulmonary valve (Figure 13). This method compares well to quantification by comparing ventricular stroke volumes [59], correlates with echocardiographic parameters [62], and a regurgitant fraction ≥40% has been considered severe [59]. Measuring pulmonary flow is especially susceptible to the problems of background flow offset errors however [25], and attempts should be made to minimise these wherever possible. This includes choosing image planes close to the transverse view (where appropriate), non-breath-hold flow sequences, and background correction where possible. Accurate measurement of RV volumes and function are particularly important, and can help guide the timing of valve replacement [60,63]. Excess RV volume loading can also be inferred from abnormal diastolic motion of the ventricular septum towards the LV in diastole, best appreciated on short axis ventricular cine images. The optimal CMR thresholds for recommending surgery are uncertain, but in adults with severe chronic PR following tetralogy of Fallot repair, a recent retrospective study noted that an RV end-diastolic volume index < 160 mL/m^2 ^resulted in a greater chance of normalisation of RV dimensions after pulmonic valve replacement [64]. Studies to assess the optimal clinical use of CMR quantification and the optimal thresholds for intervention are on-going, and may ultimately reduce the long term RV dysfunction that can result [60,65].

#### Percutaneous pulmonary valve replacement

Percutaneous pulmonary valve replacement using a stent-valve is increasing in popularity, and accurate sizing and anatomy of the pulmonary outflow tract is important for determining suitable patients [66,67]. CMR provides the required detail, in addition to accurate assessment of the valve lesion itself, and is invaluable in the assessment of patients for this procedure [66]. Despite the metal content of the stents, follow-up flow imaging can still occur above & below the stent, and some newer nitinol stents can allow flow assessment within the stent [68], though the accuracy is more uncertain.

### The tricuspid valve

#### Tricuspid regurgitation

**Figure 14:**
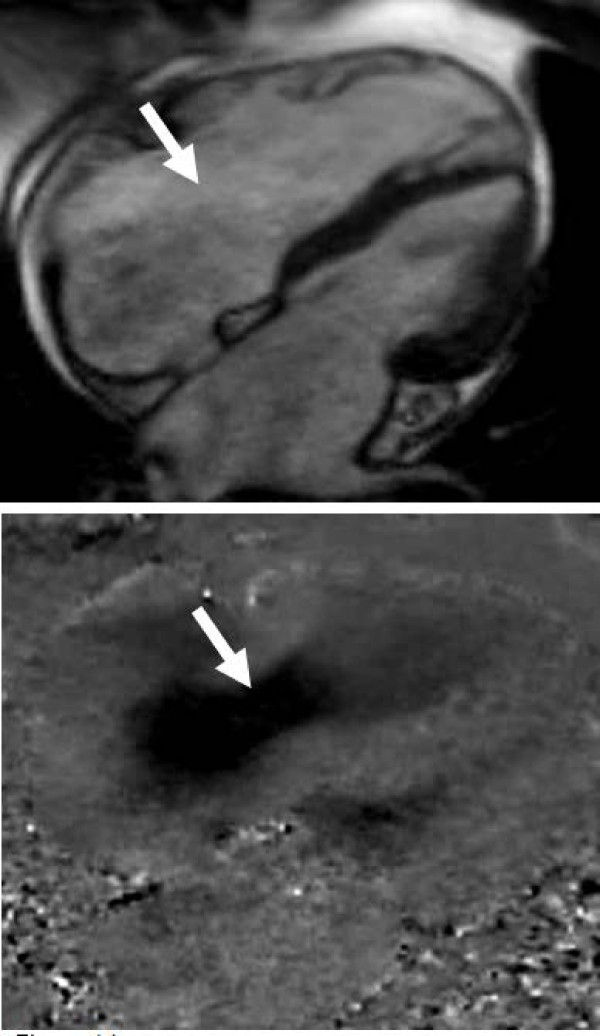
**Severe tricuspid regurgitation seen in the HLA view**. In the SSFP cine (top), there is little regurgitation seen due to the minimal turbulence from the jet. Bottom: in-plane velocity mapping sequence in the same position demonstrating the wide jet of torrential regurgitation (arrowed).

**Figure 15:**
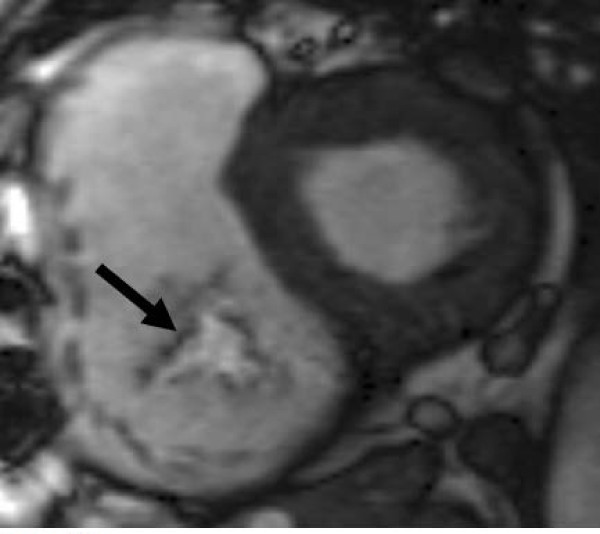
**Tricuspid regurgitation; modified short axis cine view in systole, positioned through the tricuspid valve tips**. The large regurgitant orifice is easily visualized in this case (arrowed). In some cases, through-plane velocity mapping may give clearer delineation of flow through the orifice.

CMR offers similar capabilities for the assessment of tricuspid regurgitation (TR) as for mitral regurgitation. SSFP cine sequences are used to visualise the anatomy and function of the leaflets. The horizontal long axis view provides a good overview, but multiple contiguous transverse images through the valve can often provide additional useful information, particularly for abnormal leaflet morphology such as in Ebstein's anomaly. Visualising the jet is difficult on SSFP sequences due to the lower shear and turbulence, but qualitative assessment of the TR jet can be achieved with in-plane velocity mapping in a long axis RV view (Figure 14). Wider jets (especially > 7 mm at the vena contracta) suggest more severe tricuspid regurgitation. The regurgitant orifice can sometimes be assessed directly, in a similar fashion to mitral regurgitation, with a cine image through the leaflet tips in systole (Figure 15). Through-plane velocity mapping in this plane can also aid in visualising the size of the regurgitant orifice by visualising the flow jet in cross section. This allows assessment of the regurgitant orifice area, though thresholds for guiding severity grading are not yet available. The 'diameter' of the regurgitant orifice can be assessed (as per echo protocols) but this is only an approximate guide as the regurgitant orifice is often non-circular. Again, this may be inferior to quantification of the regurgitation.

Quantification can be achieved using pulmonary flow measurement (as acquired for pulmonary regurgitation), combined with RV stroke volume to calculate the regurgitant volume (= RV stroke volume - pulmonary forward flow) and the regurgitant fraction (TR/RV stroke volumes × 100%) [69], in much the same way as for mitral regurgitation. The same limitations apply, as combination MR techniques are used to calculate regurgitation quantity, and care is required with both the flow sequence and RV contouring, which can be especially difficult. Accuracy of the flow sequence in particular is also reduced in very irregular rhythms - not uncommon with TR. Quantifying the TR can also be assessed using the difference in ventricular stroke volumes if only a single valve leak is present [70], with the same issues about the need for accurate contour placement as in MR.

Patients with abnormal placement of the tricuspid valve (Ebstein's anomaly) often present a challenge for assessing true RV volumes & function, as well as the extent of tricuspid regurgitation, due to the difficulty of identifying the true ventricle on short axis images. Good post-processing software which allows identification of the valve position in the long axis views can help considerably with this, and CMR can produce accurate assessments which aid management [71,72].

#### Tricuspid stenosis

Although extremely rare, and not routinely assessed with CMR, this valve lesion can be examined when required. Valve area can be measured by placement of an image slice through the valve tips in diastole, as for mitral stenosis, and forward velocity through the valve can be measured, though this parameter is perhaps less useful.

## Multiple valve lesions

CMR can be used to assess patients with multiple valve lesions, obtaining a detailed assessment of the severity of each component whether these occur in the same valve (i.e. mixed valve disease) or in different valves. As an extreme example, a patient with both mixed aortic and mixed mitral valve disease could have the opening area of each valve measured by direct planimetry with cine imaging to assess stenosis, the aortic regurgitation quantified from the diastolic (regurgitant) flow above the aortic valve and the mitral regurgitation quantified by subtracting the systolic (forward) flow above the aortic valve from the LV stroke volume. LV volumes and function would also be assessed. In this way, a comprehensive assessment can be undertaken.

## Prosthetic valves

**Figure 16:**
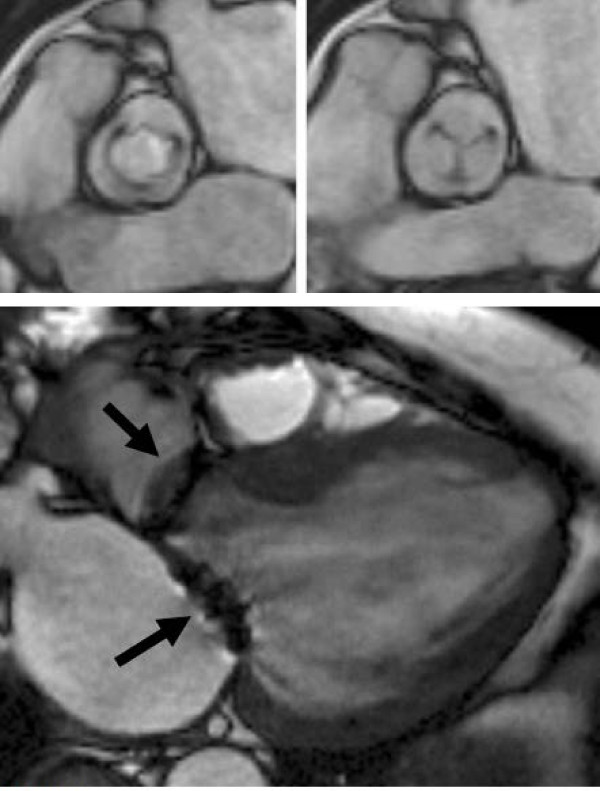
**Prosthetic valves with CMR**. Top: Carbomedics bioprosthetic valve in the aortic position in systole (left) and diastole (right). Bottom: ATS bi-leaflet tilting disc valves in both aortic and mitral positions (arrowed).

**Figure 17:**
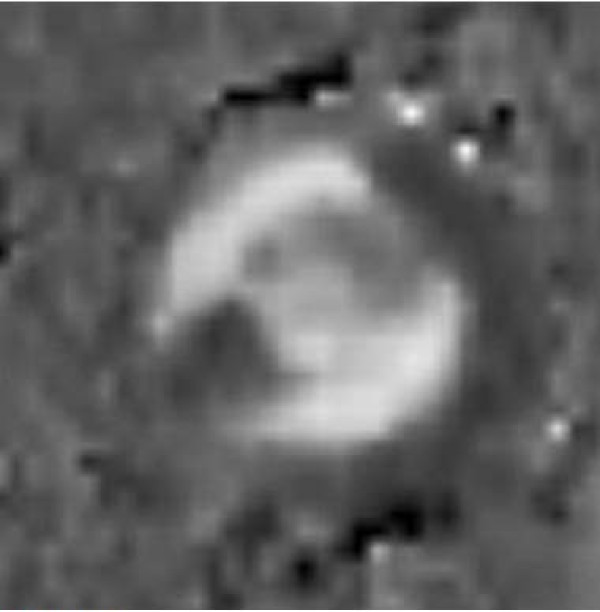
**Through-plane velocity mapping above a prosthetic aortic valve (bi-leaflet tilting disc type)**. The characteristic flow pattern of two crescents and a bridging 'strut' can be seen, reminiscent of a Star Wars™ TIE fighter viewed from the front.

Despite perceived contraindications, all prosthetic heart valves are safe in the MR scanner at 1.5 T and the vast majority are safe at 3T (not all have been tested) [73,74]. Even for mildly ferromagnetic valves, the forces exerted on the valve by the scanner are negligible when compared to those occurring with each heart-beat. Most valves produce an artefact, though the size is variable - bi-leaflet tilting discs have a smaller artefact than older ball & cage valves, though some bioprosthetic valves have significant amounts of metal in the frame which can produce a larger artefact. Prosthetic heart valves can even be assessed using CMR, including flow patterns and anatomy around the valve [75,76] (Figure 16). Some bioprosthetic valves can have the opening of the leaflets assessed by SSFP cine imaging with good accuracy [77], though not all are amenable to this. The leaflet motion of mechanical valves is not usually amenable to CMR visualisation due to the considerable artefact and the lack of signal from the valve leaflets. However, reasonable visualisation of leaflet motion can sometimes be obtained in some tilting disc valves with careful image positioning perpendicular to the leaflet hinge line. The motion of the discs may be seen from the moving signal void, but this technique is far less precise than x-ray fluoroscopy. The flow pattern of prosthetic valves can however usually be assessed using through-plane velocity mapping, with the image slice placed downstream of the signal void artefact. Bioprosthetic valves have a central flow jet similar to native aortic valves, while bi-leaflet tilting disc valves have a characteristic flow pattern (Figure 17), reminiscent of the profile of a Star Wars™ TIE (Twin Ion Engine) fighter.

Prosthetic valve dysfunction can also be assessed. If one leaflet of a bi-leaflet tilting disc valve fails to open properly, one side of the flow pattern will be missing and this may be identified with CMR. Paraprosthetic leaks can be visualised with careful image positioning, and can even be quantified with careful through-plane velocity mapping. While less conventional than other imaging modalities, these techniques can be extremely helpful in selected patients. CMR is also particularly good for assessing the anatomy of the aortic root around the valve, including grafts and valve-graft conduits, and any dissection or false aneurysm that may be present.

## Limitations

### Arrhythmias

Irregular cardiac rhythms degrade image quality, which affects the assessment of ventricular function, though the effect on this is usually small. The accuracy of flow measurement can also be reduced [54] as flow sequences are acquired over several cardiac cycles (typically 10-12), with data acquired in a complex manner, and reconstructed based on the assumption of a regular rhythm. In subjects with an irregular rhythm, the complexity of the data acquisition means that although acquired over several cardiac cycles, flow is not averaged over these, as might be expected. Where the beat-to-beat variability is small (e.g. atrial fibrillation with a controlled rate), these errors are usually not clinically significant [54]. Very irregular rhythms however (e.g. uncontrolled atrial fibrillation, multiple ventricular ectopics) can present a challenge, particularly to acquiring accurate flow data. Intelligent planning of the timing of data acquisition to the ECG can offset some of the problems, but some patients still present a challenge and caution should be exercised in interpreting the flow data in these. Cine imaging may be more reliable as it is more amenable to intelligent ECG gating and is less affected by arrhythmias, particularly during systole. Ventricular volumes vary with differing heart rates due to differential filling, and these physiological changes do of course still occur, and result in slight blurring of the myocardial borders. The result is an approximate averaging of the volumes over the several cardiac cycles of image acquisition, which is usually acceptable. Flow imaging may be improved with the newer ECG gating techniques for arrhythmias, which typically omit cardiac cycles outside a defined range.

### Haemodynamic assessment

One limitation that remains with CMR is the inability to measure directly the pressure inside a vessel or cardiac chamber - a limitation of all imaging modalities. As for echocardiography, CMR can measure velocity across a stenosis and derive a pressure drop from this, but absolute pressure quantification remains elusive. Cardiac catheterisation remains the most accurate method for assessing this. One paper has identified how CMR may indirectly indicate pressure, by examining the complex flow patterns in the pulmonary artery using 3-dimensional flow imaging [78]. The paper suggested that pulmonary pressure (measured invasively) was strongly linked with the type of flow pattern in the main pulmonary artery. The data requires further validation but indicates the novel approaches to assessment that CMR may bring in the future.

## Conclusions

CMR can provide a comprehensive assessment of valvular heart disease, including quantification of valve regurgitation and other flows, and accurate cardiac volumes and mass for assessing the effect on both ventricles. Combined with the ability to image all areas of the heart (including difficult areas such as the right ventricle and pulmonary valve), it is an excellent adjunct to echocardiography for investigating patients with valve disease. Further studies of clinical outcome, using quantitative CMR data to guide management, are needed to enhance it as a strong tool for guiding clinical practice.

## Competing interests

The author declares that they have no competing interests.

## Author's information

SGM has 15 year's experience of all areas of cardiovascular magnetic resonance, in addition to many years of echocardiography and clinical cardiology. He is the clinical lead for cardiac imaging in a major tertiary centre university hospital in Oxford, UK, and is part of Prof. Stefan Neubauer's acclaimed CMR research group. His clinical and research interests focus on valvular and aortic disease.
